# Pre-incarceration police harassment, drug addiction and HIV risk behaviours among prisoners in Kyrgyzstan and Azerbaijan: results from a nationally representative cross-sectional study

**DOI:** 10.7448/IAS.19.4.20880

**Published:** 2016-07-18

**Authors:** Maxim Polonsky, Lyuba Azbel, Martin P Wegman, Jacob M Izenberg, Chethan Bachireddy, Jeffrey A Wickersham, Sergii Dvoriak, Frederick L Altice

**Affiliations:** 1 Section of Infectious Diseases, Yale University School of Medicine, New Haven, CT, USA; 2 London School of Hygiene and Tropical Medicine, London, UK; 3 Department of Epidemiology, University of Florida Gainesville, FL, USA; 4 Department of Health Outcomes and Policy, University of Florida, Gainesville, FL, USA; 5 Department of Psychiatry, University of California San Francisco School of Medicine, San Francisco, CA, USA; 6 Department of Medicine, Harvard Medical School, Brigham and Women’s Hospital Boston, MA, USA; 7 Ukrainian Institute on Public Health Policy, Kyiv, Ukraine; 8 Division of Epidemiology of Microbial Diseases, Yale University School of Public Health, New Haven, CT, USA; 9 Centre of Excellence on Research in AIDS (CERiA), University of Malaya, Kuala Lumpur, Malaysia

**Keywords:** prisoners, police harassment, Azerbaijan, Kyrgyzstan, addiction severity, HIV risk behaviours, drug injection

## Abstract

**Introduction:**

The expanding HIV epidemic in Azerbaijan and Kyrgyzstan is concentrated among people who inject drugs (PWID), who comprise a third of prisoners there. Detention of PWID is common but its impact on health has not been previously studied in the region. We aimed to understand the relationship between official and unofficial (police harassment) detention of PWID and HIV risk behaviours.

**Methods:**

In a nationally representative cross-sectional study, soon-to-be released prisoners in Kyrgyzstan (*N*=368) and Azerbaijan (*N*=510) completed standardized health assessment surveys. After identifying correlated variables through bivariate testing, we built multi-group path models with pre-incarceration official and unofficial detention as exogenous variables and pre-incarceration composite HIV risk as an endogenous variable, controlling for potential confounders and estimating indirect effects.

**Results:**

Overall, 463 (51%) prisoners reported at least one detention in the year before incarceration with an average of 1.3 detentions in that period. Unofficial detentions (13%) were less common than official detentions (41%). Optimal model fit was achieved (*X*^2^=5.83, *p=*0.44; Goodness of Fit Index (GFI) GFI=0.99; Comparative Fit Index (CFI) CFI=1.00; Root Mean Square Error of Approximation (RMSEA) RMSEA=0.00; PCLOSE=0.98) when unofficial detention had an indirect effect on HIV risk, mediated by drug addiction severity, with more detentions associated with higher addiction severity, which in turn correlated with increased HIV risk. The final model explained 35% of the variance in the outcome. The effect was maintained for both countries, but stronger for Kyrgyzstan. The model also holds for Kyrgyzstan using unique data on within-prison drug injection as the outcome, which was frequent in prisoners there.

**Conclusions:**

Detention by police is a strong correlate of addiction severity, which mediates its effect on HIV risk behaviour. This pattern suggests that police may target drug users and that such harassment may result in an increase in HIV risk-taking behaviours, primarily because of the continued drug use within prisons. These findings highlight the important negative role that police play in the HIV epidemic response and point to the urgent need for interventions to reduce police harassment, in parallel with interventions to reduce HIV transmission within and outside of prison.

## Introduction

Despite marked declines globally, HIV incidence and mortality continue to rise in Central Asia and the Southern Caucuses – two neighbouring regions comprised of former Soviet Union (FSU) states [1]. People who inject drugs (PWID) are responsible for approximately 70% of new HIV infections in Central Asia [2]. The scenario in the Southern Caucuses is more mixed, but injection accounts for over half of all HIV transmission in Azerbaijan and Georgia, two of the region's three countries [3]. Kyrgyzstan and Azerbaijan are representative of Central Asia and the Southern Caucuses, respectively, with epidemics that are highly concentrated among at-risk populations, in particular PWID [4]. At 32.4%, Kyrgyzstan has the highest upper estimate of HIV prevalence in PWID in the region. In Azerbaijan, HIV prevalence among PWID ranges between 19 and 24% [5]. Throughout Central Asia and the Southern Caucuses, opioids are the primary drugs injected, likely due to their availability from heroin trafficking originating in nearby Afghanistan [6].

One of the major global challenges to addressing the HIV epidemic among PWID has been the legal environment facing PWID, specifically the criminalization of drug possession, use and addiction [7–11]. Policing practices play a major role in constructing this “risk environment” [10,12] promoting risky behaviour such as rushed injection [13,14], overdose [15], use of non-sterile syringes [13,16], and undermining uptake of and adherence to increasingly available evidence-based options for the prevention of HIV transmission among PWID, such as needle-syringe programmes (NSP), opioid agonist therapies (OAT) with methadone or buprenorphine, and antiretroviral therapy (ART) [17–20]. Policing practices that affect PWID include targeted enforcement at treatment facilities [21,22], intimidation of providers [23,24] and syringe confiscation [25]. Particularly damaging may be unofficial detention of PWID (involving no formal charges and often undertaken outside the scope of the law), which can be conceptualized as a form of police harassment. A recent study in Ukraine found this practice to be common, often resulting in opioid withdrawal and prolonged interruptions of ART and OAT [17].

In Central Asia and the Southern Caucuses, arrests and detentions of PWID, both official and unofficial, are common [10,26–29]. Neither the prevalence of unofficial detention of PWID nor its impact on health, however, has been examined in these regions. This study aims to understand the prevalence of pre-incarceration police detention among nationally representative incarcerated PWID, as well as the comparative impact of official and unofficial detention on PWID HIV risk behaviour, such as unprotected sex and use of non-sterile injection equipment in Kyrgyzstan and Azerbaijan. The focal research question here is whether police target PWID and whether such targeting is associated with increased HIV risk-taking behaviours.

## Methods

The sampling, inclusion criteria and survey methods with survey content have been previously described [29,30]. Briefly, a nationally representative biobehavioural health survey of prisoners within six months of release was conducted from February to November 2014. Eligible adult prisoners were randomly sampled from 8 prisons in Kyrgyzstan (*N*=368) and from 13 prisons in Azerbaijan (*N*=510). They completed confidential, self-administered surveys assessing HIV risk, health status and criminal justice involvement using audio-computer-assisted self-survey instruments (ACASI) on touch-screen laptop computers [31] to ensure anonymity, minimize social desirability bias, and facilitate ethical principles of conducting research with prison populations [32]. Participants were randomly selected from all sentenced prisoners within six months of release in non-specialized facilities in both countries using a stratified random sampling scheme [33] previously validated in Eastern Europe and Central Asia [29,30,34]. Inclusion criteria for participation included (1) ≥18 years, (2) currently serving a sentence in a non-specialized facility and (3) scheduled to be released within six months. Specialized facilities (juvenile detention and hospital prisons) and pre-trial detention centres were not included. Experienced research assistants (RAs) from local NGOs that work with prisoners underwent extensive training on study methods and confidentiality procedures. They used a random assignment chart to select participants who were informed by prison staff that they were randomly selected for participation in a voluntary and anonymous health study. The enrolment was kept proportional to the number of prisoners within six months of release in each country (50% for Azerbaijan and 40% for Kyrgyzstan). From an estimated 1037 inmates in non-specialized facilities meeting eligibility criteria in Azerbaijan, 535 were selected, and 25 (4.7%) refused participation. The eligible sample size in Kyrgyzstan was 938 inmates, and among 381 selected participants, 13 (3.4%) did not provide informed consent.

### Study measures

Surveys were originally constructed in English, translated into Russian, Azeri and Kyrgyz languages, back translated into English [35], reviewed by bilingual researchers and piloted to ensure clarity, quality and respondents’ comprehension. In addition to demographic characteristics, the 10-item Clinical Epidemiological Survey of Depression (CES-D 10) [36]; Zung anxiety scale [37]; and WHO's Alcohol Use Disorders Inventory Test (AUDIT) [38] were included. The Addiction Severity Index – Lite Version [39] was used to measure addiction severity.

HIV risk behaviours were measured using an adapted set of items from NIDA's Risk Behavior Assessment (RBA) addressing sexual and drug risk-taking behaviours in the 30-day period prior to the arrest that resulted in the current incarceration. Sexual risk was measured by frequency of unprotected sex events, and drug risk was measured by the number of injection days multiplied by the average number of injections per day using non-sterile injection equipment. The sum of these items formed a composite measure, *HIV Risk* [40]. Noteworthy, in Kyrgyzstan, due to more lenient regulations, relative to the ones that exist in Azerbaijan, which did not require reporting drug use to the prison department, questions about within-prison injection-related risk behaviours were assessed during the survey. This provided a unique opportunity to measure current within-prison drug injection (WPDI) [30]. WPDI was measured as a binary response to whether or not injection occurred during the current incarceration. Social support was measured using the Multidimensional Scale of Perceived Social Support [41].

### Detention measures

Detention was defined as an event of being detained in police lock-up the year before incarceration when that event did not lead to the current incarceration. Using previously defined measures [17], detention history consisted of two measures asking respondents to report the number of *official* and *unofficial* detentions in the year before the current incarceration. An official detention was defined as detention accompanied by formal charges, whereas an unofficial detention was defined as detention not accompanied by a charge (e.g. drug possession, theft). Based on previous research in the region, unofficial detentions are considered a form of police harassment [17]. The sum of these two items served as the composite measure of *detention*. Further, respondents were asked about each of the following adverse effects during their unofficial and/or official detention: symptoms of abstinence syndrome (withdrawal from opioids), interruption of HIV and OAT medications for more than 24 hours, and inability to see a medical provider if needed. Respondents were also asked whether their drug use, access to OAT, HIV or TB treatment was used to extract a confession, and whether they were stopped, searched, held or arrested while traveling to or from a NSP site.

### Data analysis

To guide our analysis, we hypothesized that police may selectively target PWID and that such harassment practices may translate into increased HIV risk behaviours. Hence, the focal interest in the analyses was the relative association of official and unofficial detention with drug addiction severity and with the outcomes: HIV risk behaviours and WPDI. For the cross-cultural analysis between the two countries, the primary outcome measure was HIV risk, while for the Kyrgyzstan sub-analysis, the primary outcome was current WPDI, which measures present time injection and therefore provides a unique opportunity to establish temporal ordering in our cross-sectional data.

SPSS, version 22, was used to compute correlation and multiple regressions to assess multivariate relationships among the variables. Non-parametric *χ*^2^ tests and independent sample *t*-tests were utilized to measure differences between detained and not detained participants on each of the described measures. The structural equation modelling programme AMOS.22 was utilized to perform a multi-group path analysis. To calculate indirect effects and investigate potential mediating relationships among the variables in the model, we used the AMOS bootstrapping procedure [42], a recommended analytic strategy for avoiding measurement error and underestimation of the mediation significance [43].

### Ethics statement

Institutional Review Boards at Yale University, the Ukrainian Institute on Public Health Policy and the Kyrgyzstan Ministry of Health approved the study. Further ethical and safety assurances were provided by the Office for Human Research Protections (OHRP) in accordance with 45 CFR 46.305(c) “Prisoner Research Certification” requirements. Participants provided written informed consent prior to study participation.

## Results

Tables 1 and 2 provide descriptive statistics for detained and not detained prisoners in the year before their current incarceration in Kyrgyzstan and Azerbaijan, respectively. The prevalence of recent detention was 51.5% in Kyrgyzstan and 34% in Azerbaijan. In both countries, detained participants reported higher average prison sentences, more years in prison, lower age of first incarceration and higher frequency of unprotected sex relative to prisoners who had not been detained. In Kyrgyzstan, injection within the current incarceration was higher among detained than not detained prisoners. In Azerbaijan, detained prisoners reported higher instances of injection and polysubstance use, as well as higher levels of social support. Table 3 provides details on experiences associated with official and unofficial detention among detained prisoners in both countries.

**Table 1:** Comparison of detained and not detained participant characteristics in Kyrgyzstan (*N*=355)

Characteristics	Valid *N*	Total *n* (%)	Not detained *n* (%)	Detained *n* (%)	p*
Mean age (SD)	352	37.6 (11.3)	37.3 (11.2)	38.0 (11.4)	0.561
Male gender	353	273 (77.3)	107 (68.6)	166 (84.3)	**0.001**
Mean prison sentences before this incarceration (SD)	220	3.5 (2.2)	1.69 (2.2)	2.53 (2.6)	**0.001**
Mean lifetime years in prison (SD)	353	8.2 (6.9)	6.5 (5.5)	9.6 (7.5)	**<0.001**
Mean age of first incarceration (SD)	353	26.2 (11.2)	29.0 (9.9)	24.0 (12.1)	**<0.001**
Alcohol dependence in the year before this incarceration	352	150 (42.6)	58 (37.2)	92 (46.9)	0.082
ASI drug use composite score (SD)	350	0.08 (0.09)	0.07 (0.01)	0.08 (0.09)	0.177
Injected during current incarceration	353	69 (19.3)	21 (13.5)	47 (23.9)	**0.015**
Sexual intercourse without condom in 30 days before incarceration	352	175 (49.7)	72 (46.2)	103 (52.6)	0.139
Mean episodes (unprotected sex)	175	4.2 (7.7)	3.0 (6.5)	5.1 (8.4)	**0.013**
Moderate to severe symptoms of depression	353	118 (33.4)	58 (37.2)	60 (30.5)	0.212
Social support	355	2.8 (1.0)	2.8 (0.9)	2.8 (1.0)	0.654
Anxiety disorder	353	22 (6.2)	9 (5.8)	13 (6.6)	0.827

* Compares detained vs. not detained. Significance defined as *p* < 0.05, and marked in bold.

**Table 2:** Comparison of detained and not detained participant characteristics in Azerbaijan (*N*=496)

Characteristics	Valid *N*	Total. *n* (%)	Not detained *n* (%)	Detained *n* (%)	p*
Mean age (SD)	496	38.2 (8.9)	38.3 (8.7)	37.6 (9.1)	0.404
Male gender	496	447 (90.1)	319 (97.6)	128 (75.7)	**<0.001**
Mean prison sentences before this incarceration (SD)	152	1.6 (0.7)	1.5 (0.7)	1.8 (0.7)	**0.005**
Mean lifetime years in prison (SD)	496	4.6 (3.8)	3.7 (0.2)	3.8 (0.3)	**0.002**
Mean age of first incarceration (SD)	487	30.1 (8.8)	30.7 (8.8)	28.8 (8.6)	**0.023**
Alcohol dependence in the year before this incarceration	496	50 (10.2)	29 (8.9)	21 (12.7)	0.209
ASI drug use composite score (SD)	482	0.06 (0.04)	0.06 (0.04)	0.07 (0.05)	0.116
Ever injected drugs	496	157 (31.7)	100 (30.6)	57 (33.7)	0.478
Substance use in 30 days before this incarceration	466	166 (35.6)	105 (32.8)	61 (41.8)	0.076
30 or more injections	131	24 (18.3)	11 (12.6)	13 (29.5)	**0.030**
Used more than one substance	496	38 (7.7)	16 (4.9)	22 (13.0)	**0.002**
Sexual intercourse without condom in 30 days before incarceration	495	176 (35.5)	102 (31.3)	74 (43.8)	**0.007**
Mean episodes (unprotected sex)	176	16.7 (13.2)	0.06 (0.04)	0.07 (0.05)	0.623
Moderate to severe symptoms of depression	491	126 (25.4)	90 (27.7)	36 (21.7)	0.157
Social support (SD)	496	3.1 (1.2)	2.9 (1.2)	3.6 (0.9)	**<0.001**
Anxiety disorder	490	23 (4.7)	17 (5.2)	6 (3.6)	0.504

* Compares detained vs. not detained. Significance defined as *p*<0.05, and marked in bold.

**Table 3:** Experiences associated with police detention among prisoners in Kyrgyzstan and Azerbaijan, accounting for official and unofficial detention

Detentions and related events (year before current incarceration)	Valid *N*		Total *n* (%)		Official detention^a^ *n* (%)		Unofficial detention^a^ *n* (%)	
			
Country	KYR	AZ	KYR	AZ	KYR	AZ	KYR	AZ
Detained	355	496	183 (51.5)	169 (33.9)	182 (51.5)	169 (33.9)	76 (21.4)	35 (7.0)
Mean number (SD)	352	496	3.1 (4.7)	2.4 (2.3)	1.4 (1.3)	1.8 (1.3)	4.8 (6.2)	1.5 (1.0)
Experienced withdrawal during a detention (among those using drugs at time of detention)	155	91	27 (17.4)	25 (27.5)	24 (17.0)	22 (25.6)	15 (21.7)	9 (39.1)
ART interrupted during detention (among those detained while on ART)	6	0	2 (33.3)	0	2 (33.3)	0	2 (50.0)	0
OAT interrupted during detention (among those detained while on OAT)	14	0	6 (42.9)	0	6 (46.2)	0	4 (44.4)	0
Had restricted access to ART, TB medication and/or OAT used as a means to extract a confession during a detention (among those on ART, TB, OAT)	78	55	20 (25.6)	1 (0.2)	16 (25.8)	0	13 (28.9)	1 (7.1)

a Percent of those reporting for whom it is applicable. KYR, Kyrgyzstan; AZ, Azerbaijan; ART, antiretroviral therapy; TB, tuberculosis; OAT, opioid agonist therapy; SD, standard deviation.

Importantly, there was no difference between PWID and people who did not inject drugs in their experiences with official detention (*t*>1.32, *p*>0.18 for both countries), but there was a difference in reports of unofficial detention. Specifically, PWID experienced significantly higher unofficial detention by police relative to people who did not inject drugs in Azerbaijan (*M*=0.22, SD=0.72 vs. 0.05, SD=0.26, *t*=2.76, *p*=0.01) but not in Kyrgyzstan (*t*=1.83, *p*=0.07).

### Effects of detention in Azerbaijan and Kyrgyzstan

The inter-correlation between official and unofficial detention was weak, but significant (*r*=0.19), and both detention variables differed in significance and magnitude in their association with drug addiction severity and HIV risk (Table 4). To explore the relative effects of detention on HIV risk-taking and investigate potential mediating relationships among the variables identified as significant correlates through bivariate testing while also accounting for moderating impact of each country, we performed a multi-group path analysis with official and unofficial detention as exogenous variables, addiction severity as a mediator, and composite HIV risk as an endogenous variable. We controlled for depression, anxiety, social support and the presence of alcohol use disorders and estimated indirect effects via bootstrapping procedures, while step-wise eliminating insignificant paths and “hanging” variables.

**Table 4:** Correlations among the variables used in the path analysis

Measure	1	2	3	4	5	6	7
1. Official detention	–						
2. Unofficial detention	0.19*	–					
3. Addiction severity	0.08*	0.19*	–				
4. HIV risk	0.01	0.12*	0.55*	–			
5. Anxiety	−0.10*	0.03	0.11*	0.06	–		
6. Depression	−0.03	0.12*	0.12*	−0.02	0.58*	–	
7. Alcohol use disorder	0.04	0.20*	0.20*	0.09*	0.05	0.24*	–
8. Social Support	0.12*	−0.04	0.02	0.21*	−0.05	−0.17*	−0.03

* *p*<0.01.

**Figure 1:**
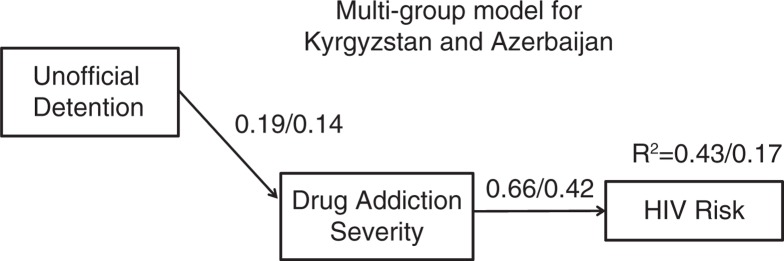
Multi-group results for mediation analysis. Country moderated the relationship between unofficial detention and HIV risk: Direct effect from unofficial detention to HIV risk was significant (0.17) for KYR and not significant for AZ (0.02). Overall model fit: *X*^2^=.435; df=2; *p*=0.805; Root Mean Square Error of Approximation (RMSEA) RMSEA=0.000 (PCLOSE=0.911); Comparative Fit Index (CFI) CFI=1.00; Goodness of Fit Index (GFI) GFI=0.999. Multi-group results in figure correspond to Table 2. The results (of multiple regression) showed that country moderated the relationship between unofficial detention and HIV risk: Direct effect from unofficial detention to HIV risk was significant (0.17) for KYR and not significant for AZ (0.02). KYR, Kyrgyzstan; AZ, Azerbaijan.

Optimal model fit was achieved (*X*^2^=5.83, *p*=0.44; GFI=0.99; CFI=1.00; RMSEA=0.00; PCLOSE=0.98) when unofficial detention had an indirect effect on HIV risk, fully mediated by drug addiction severity, with more detentions associated with higher drug addiction severity – in turn correlating with increased HIV risk-taking behaviours. There were two significant covariates in the model (Table 5). The multi-group model with an identical path structure was a good fit to the data as well (*X*^2^=1.98, *p*=0.37; GFI=0.99; CFI=1.00; RMSEA=0.00; PCLOSE=0.84). Our final aggregate model is presented in Figure 1, and the multi-group moderated mediation results with indirect, direct and total effects for both countries presented in Table 6. For both countries, addiction severity fully mediated the effect of unofficial detention on HIV risk, whereby unofficial detention was positively associated with addiction severity that in turn was positively associated with HIV risk-taking behaviours. Both the association between addiction severity and HIV risk, and the indirect effect from unofficial detention to HIV risk, were higher in Kyrgyzstan. The final model explained 43% of the variance in the outcome in Kyrgyzstan and 17% in Azerbaijan. Our results confirm and further clarify the hypothesized relationship between detention and HIV risk-taking behaviours.

**Table 5:** Significant covariates in the final path model^a^

Control variable	Criterion variable	B (SE)	C.R.	Beta
Anxiety	Addiction severity	0.01 (0.00)	3.25	0.11
Social support	HIV risk	3.5 (0.48)	7.23	0.20

a All coefficients are significant at *p*<0.01.

**Table 6:** Direct, indirect and total effects among the variables in the multi-group model

Variables Predictor	Criterion Country	Direct effects Beta (SE)		Indirect effects Beta (SE)		Total effects Beta (SE)	
		
		KYR	AZ	KYR	AZ	KYR	AZ
Unofficial detention	Addiction severity	0.19^a^ (0.08)	0.14 (0.06)	–	–	0.19 (0.08)	0.14 (0.06)
	HIV risk	–	–	0.12 (0.05)*	0.06 (0.03)*	0.12 (0.05)	0.06 (0.03)*
Addiction severity	HIV risk	0.65 (0.06)*	0.42 (0.07)*	–	–	0.65 (0.06)	0.42 (0.07)*

a All coefficients in the model are significant at *p*<0.01.

* Significant difference between two countries at *p*<0.01. KYR, Kyrgyzstan; AZ, Azerbaijan.

### Effects of detention on within prison drug injection in Kyrgyzstan

Current WPDI was measured only among our participants in Kyrgyzstan, but it is a crucial outcome variable to consider in order to further confirm and clarify the relationship between police detention and HIV risk-taking behaviours within prison, which is an especially high risk behaviour. WPDI is a behavioural outcome that measures current injection within the high-risk prison environment and therefore introduces temporal order to our self-reported cross-sectional data. Arguably, if our results from the multi-group analysis reported above are replicated with a conceptually stronger outcome measure, the generalizability of the mediated relationship between police detention and HIV risk behaviours would gain in credibility.

**Figure 2:**
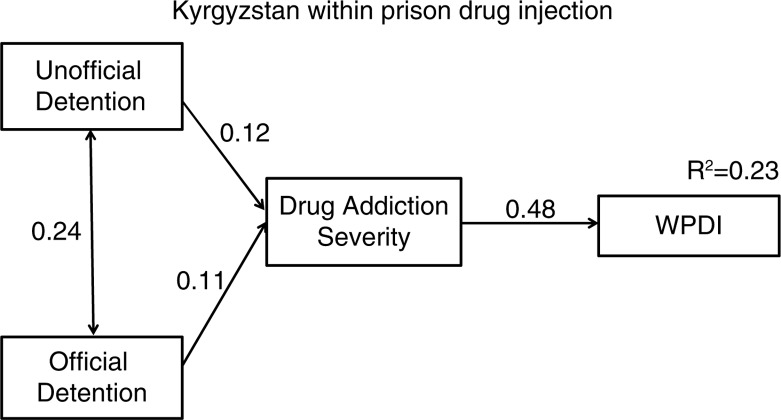
Path model for unofficial and official detention effects on within-prison drug injection (WPDI) mediated by addiction severity. All paths are significant at *p*<0.01. Indirect effects were tested via AMOS bootstrapping procedure with 4000 bootstrap samples and bias-corrected confidence intervals. Overall model fit: *X*^2^=0.435; df=2; *p=*0.805; Root Mean Square Error of Approximation (RMSEA) RMSEA=0.000 (PCLOSE=0.911); Comparative Fit Index (CFI) CFI=1.00; Goodness of Fit Index (GFI) GFI=0.999. Both official and unofficial detention for KYR subset only, due to current WPDI. Standardized bootstrap indirect effects. Unofficial to WPDI=0.06, *p*=0.05. Official to WPDI=0.06, *p*=0.05.

Thus, we ran a similar path model to the one we reported for both countries: with detention variables as predictors, addiction severity as a mediator, and WPDI as the outcome measure of HIV risk behavior. The final model presented in Figure 2 for the Kyrgyzstan sub-analysis is a full mediation model (*X*^2^=0.44, *p*=0.81; GFI=1.00; CFI=1.00; RMSEA=0.00; PCLOSE=0.91) that shows addiction severity mediating the effect of detention on WPDI. Official and unofficial detention were both significant correlates of addiction severity with a similar magnitude, and had equal indirect effects on WPDI (see Table 7). Because WPDI is a dichotomous outcome measure, we followed a statistical solution for mediation analysis with dichotomous variables, recommended by MacKinnon and Dwyer [44,45].

**Table 7:** Direct, indirect and total effects among the variables in the WPDI^a^ model

Variables		Direct effects	Indirect effects	Total effects
Predictor	Criterion	Beta (SE)	Beta (SE)	Beta (SE)
Unofficial detention	Addiction severity	0.12^b^ (0.07)	–	0.12 (0.072)
	WPDI	–	0.06 (0.04)	0.06 (0.04)
Official detention	Addiction severity	0.11 (0.06)	–	0.11 (0.06)
	WPDI	–	0.06 (0.03)	0.06 (0.03)
Addiction severity	WPDI	0.48 (0.05)	–	0.48 (0.05)

a Within prison drug injection (WPDI). Kyrgyzstan sample only

b all coefficients in the model are significant at *p*<0.01.

## Discussion

The data presented here draw attention to the role of policing practices and police harassment in driving the spread of HIV, addressing a major structural challenge to HIV prevention in countries of the FSU in the Eastern European and Central Asian region, where HIV incidence and mortality continue to increase. Kyrgyzstan's and Azerbaijan's HIV epidemic, like those in neighbouring Eastern European and Central Asian countries, is closely intertwined with substance use and criminal sanctions against PWID [4]. PWID comprise one-third of the prison population in these two countries [29,30] and police harassment is common, but this study is the first to examine the impact of policing behaviours on negative health consequences in this region. Our results are consistent with an emerging body of literature that attests to law enforcement as a major roadblock to scaling-up HIV prevention interventions both in the region [4,27], and globally [18,46–48]. Insights drawn here, from the only scientifically rigorous biobehavioural surveys among prisoners in two FSU countries, provide the first evidence of the effect of policing on concentrating and promulgating HIV risk-taking within prisons. These findings highlight the important role that police might play in HIV prevention and point to the urgent need for changing the role of the police, including structural changes in policing practices, to reduce police harassment of PWID who spend considerable time in prison and remain the primary drivers of HIV in the region.

Our analyses disentangle the mediating and moderating relationships between police detention, addiction severity and HIV risk and demonstrate the importance of performing moderated mediation analyses to account for data complexity, as well as for revealing often surprising relationships in the data. Detention had an indirect effect on HIV risk, mediated by addiction severity, with more detentions associated with higher addiction severity – in turn correlating with increased HIV risk. This pattern suggests that police selectively target PWID with higher addiction severity. Rather than target them for arrest, police should align their practices with public health and steer them toward evidence-based treatment with methadone or buprenorphine, both of which reduce addiction severity and HIV risk-taking behaviours [49], and help avoid incarceration. Alternatively, if OAT is not available or PWID are not injecting opioids, they can encourage use of NSP, which also reduces HIV risk [50].

Moreover, these patterns hold for both Azerbaijan and Kyrgyzstan, pointing to a wider and consistent trend throughout the region. Rates of detention in our sample were high, with over half and a third of participants in Kyrgyzstan and Azerbaijan, respectively, reporting detention in the year before incarceration. Country acts as a moderator in the model and the effect of police detention on HIV risk is stronger for Kyrgyzstan, where over one-third of those accessing services reported disruption in ART, OAT or NSP access as a result of detention. This is consistent with data showing that police detention and the fear of police harassment impedes PWID's capacity for HIV risk reduction [12,51], leading to sharing of injection equipment and decreased engagement in harm reduction services.

These data are the first to draw a health distinction between unofficial (extrajudicial and therefore deemed harassment) and official (judicial and potentially with just cause) detention. While both unofficial and official detention contribute to increased HIV risk-taking behaviours, mediated by addiction severity, unofficial detention is more strongly associated with the outcome. Police harassment here is a correlate of addiction severity, which mediates its effect on HIV risk behaviour. It is well established that community policing is often inconsistent with established guidelines, interfering with harm reduction programmes and undermining health and human rights [10]. The negative health effects of unofficial detention are consistently stronger than those for official detention in Kyrgyzstan (see Table 3). The heavy-handed role of policing in the region is embedded in a historical context [27], where interventions for PWID in the Soviet Union were limited to non-evidence based and unethical forced detox, treatment with neuroleptics, labour camps, and social isolation [2]. This legacy is now evident in the harassment of PWID for possessing small amounts of drugs for personal use, and arrest of methadone patients outside of addiction treatment clinics [52]. It is no surprise, then, that police harassment of PWID, who are at heightened risk for blood-borne infections, is a structural factor contributing to HIV transmission in the community.

Our data from Kyrgyzstan are the first to provide a glimpse into the role of police harassment in promoting onward HIV transmission not only in the community, but also within the extraordinarily high-risk prison environment where injection equipment is scarce and associated with heightened transmission risk. In Kyrgyzstan, detention is fully mediated by addiction severity on current WPDI. This is especially pertinent given that WPDI is extremely common in PWID [30]. Our results suggest that police target PWID and that such harassment may result in the increase in HIV risk-taking behaviours, primarily because of the continued drug use within prisons. It is well established that treating addiction within criminal justice settings is key, including implementing OAT and effectively transitioning them to the community [4], which will not only reduce HIV transmission, but improve HIV- and non-HIV-related health outcomes [53–58]. Even though our decision to measure and compare official and unofficial detention allowed us to more closely examine the relationship between detention, addiction severity and HIV risk-taking behaviours, it is important to note that both types of detention may constitute police harassment, including those instances of official detention that resulted in the current incarceration.

Though meaningful findings were gleaned from our research, several limitations remain. First, the cross-sectional design restricts our ability to infer a causal nature of the observed relationships and limits the findings to correlations. The study's focus on distinct time periods of detention experiences and health risk behaviours, however, lessen some of these concerns by outlining a hypothesized causal mechanism that can be subsequently elucidated with longitudinal design. It is important to note that the current WPDI measure for the Kyrgyzstan sample has allowed us to address and clarify temporal ordering in our cross-sectional data. Conversely, our inability to include a similar measure in Azerbaijan due to obligations to report drug use to prison department is a limitation. Clearly, further research employing longitudinal designs that would allow establishing causality and likely result in more meaningful mediating and moderating relationships is warranted. Also, we relied on self-reported measures for several parameters, including for opioid injection, but these were validated measures and the sheer magnitude suggest that they represent conservative amounts of drug use. This could have resulted in underreporting of health risk behaviours due to social desirability bias. Self-reporting may also result in underreporting of detention experiences, although the observed high rates of detention in our study reduce this concern. Another potential limitation that may restrict interpretation and accuracy is recall bias, since participants had to report on remote pre-incarceration behaviours and experiences. Notwithstanding these limitations, our findings point to a conceivable mechanism of the effects of policing practices on the health of PWID who interface with criminal justice system and lay the foundation for future research to replicate and expand these findings, as well as for future strategies to engage police enforcement in advancing individual and public health.

## Conclusions

Given the police's role in shaping HIV transmission, it is now necessary to shift focus to best-practice implementation strategies to influence HIV prevention. While most HIV prevention has been focused on individual changes in behaviour, our data provide empirical support for the environmental influence of policing on HIV risk. PWID exist in complex risk environments where factors interact to produce drug-related harm [60]. Accordingly, successful biobehavioural interventions delivered to PWID, including OAT expansion, must address environmental factors, which can include intimidation, violent victimization, marked social stratification, and stigmatization of people with or at risk for HIV, and people who receive drug treatment and OAT in particular [60,61]. Therefore, police interaction with PWID should be harnessed and aligned with HIV prevention to implement evidence-based harm reduction practices including referral to NSPs, supervised injection sites, and OAT [10]. There is new evidence that targeted police training in Kyrgyzstan that focuses on HIV prevention is associated with improved public health knowledge [18]. Furthermore, making positive health outcomes an incentive for assessing police performance is key to increasing law enforcement's concern for health. Fostering partnerships between law enforcement and the public health sector is paramount to ensuring improved health outcomes among marginalized populations [62].

## Supplementary Material

Pre-incarceration police harassment, drug addiction and HIV risk behaviours among prisoners in Kyrgyzstan and Azerbaijan: results from a nationally representative cross-sectional study
