# Supplementary material for: Pre-incarceration police harassment, drug addiction and HIV risk behaviours among prisoners in Kyrgyzstan and Azerbaijan: results from a nationally representative cross-sectional study
Source: J Int AIDS Soc. 2016 Jul 18;19(4Suppl 3):20880. doi: 10.7448/IAS.19.4.20880 (PMC4951538; doi:10.7448/IAS.19.4.20880)
Supplement: Pre-incarceration police harassment, drug addiction and HIV risk behaviours among prisoners in Kyrgyzstan and Azerbaijan: results from a nationally representative cross-sectional study [file JIAS-19-20880-s001.pdf]

Technical Supplement

Preamble

We encourage the curious readers to consult the growing selection of books, book chapters, and seminal articles to get more exposure and familiarize themselves with the framework of structural equation modeling (SEM). SEM is a diverse class of statistical methods and analytic techniques, which include factor analysis, path analysis, and latent growth and process modeling, and is often used to estimate latent (i.e., unobserved) constructs and the nature of the relationship among them. It is important to mention from the start that our paper reports the results of path analysis, a special case of SEM, where the focus is on relationships of multiple observed or directly measured, and not latent, variables. Path models provide researchers with a more complete theoretical testing, specification, and understanding relative to traditional regression analyses. Path models allow one to examine direct, indirect, and total effects simultaneously in one model, as well as to apply some of the bootstrap resampling techniques in SEM programs, which also are able to appropriately correct for missing data problems and non-normality in the data. Essentially, however, performing multi-group path analysis is similar to running multiple regressions with interaction terms simultaneously; therefore, the results presented in the manuscript are likely to hold with less sophisticated and more common techniques.

This supplement provides more detail on our multi-group path analysis that was performed in AMOS.22. To calculate indirect effects and investigate potential mediating relationships among the variables in the model, we used the AMOS bootstrapping procedure with a bias-corrected accelerated confidence intervals,[1] a recommended analytic strategy for avoiding measurement error and underestimation of the mediation significance.[2]

Specifically, to explore the relative effects of detention on HIV risk-taking, and investigate potential mediating relationships among the variables identified as significant correlates through bivariate testing while also accounting for moderating impact of each country, we performed a multi-group path analysis with official and unofficial detention as exogenous variables, addiction severity as a mediator, and composite HIV risk as an endogenous variable. We controlled for depression, anxiety, social support, and the presence of alcohol use disorders, and estimated indirect effects via bootstrapping procedures, while step-wise eliminating insignificant paths and "hanging" variables.

As noted in the manuscript, our data is cross-sectional; nevertheless, we believe it is important to clarify the temporal ordering in the questionnaire. Detention history measures asked about experiences in the year before the current incarceration; while drug addiction and HIV risk behaviors were assessed by a set of items addressing behaviors in the 30-day period prior to the arrest that resulted in the current incarceration. Being able to measure the current within prison drug injection (WPDI) for the Kyrgyzstan sample has allowed us to further clarify and confirm temporal ordering in our cross-sectional data. Thus, the relationships between detention, addiction severity, and HIV-risk are correlational, although they follow the temporal ordering of the survey.

Measures of Model Fit

The chi-square value χ^2 (with degrees of freedom and a corresponding p value) can be used to assess whether a specified model fits the data; however, this statistic should be used with caution, as it is sensitive to sample size, and is likely to result in statistical significance with a large sample size, rejecting the null that the model fits the data.[3]

Comparative fit index (CFI)[4] is a commonly used index that involves comparing the fit of a specified *default* model against the fit of the null *independence* model that assumes no relationships among the variables.

Root Mean Square Error of Approximation (RMSEA) is a widely used index of model fit that looks at the differences between observed and predicted covariances and corrects for model complexity. A value of the RMSEA of about .05 or less typically indicates a good fit of the model in relation to the degrees of freedom, but a value of .08 or less are also adequate. The RMSEA is reported with the 90% confidence interval: LO 90 and HI 90.

PCLOSE is a p value that tests the null hypothesis that the RMSEA is no greater than .05, and if p is greater than .05, the model fit is close.

The goodness of fit index (GFI) ranges from 0 to 1, where 1 indicates a perfect fit. It is important to note that this measure is strongly affected by sample size and is reported here conventionally, as it has not lost its popularity despite the documented bias (see Kline[5]).
AGFI is adjusted GFI for the degrees of freedom, bounded by 1 above but is not bounded below by zero.

Path Diagrams

SEM is a graphical modeling methodology, so the graphical representation is essential to defining and formalizing the specification of a model. As can be seen in the path diagram below (Model 1), rectangles represent observed variables, which are the measurement constructs described under the study measures section of the manuscript. These variables are exogenous (predictors) and endogenous (criterion).

As shown in the path diagram, small circles (labeled *e*) are error terms or disturbances, which are latent influences on the constructs and reflect both variance attributable to random processes as well as the processes specific to that construct, and are usually attached to all endogenous variables in the model.[6]

Model 1. Base aggregate model with covariates for two countries combined without bootstrap procedure.

Note: variable description “Drugs” refers to addiction severity and “HIVoutcome” refers to HIV risk throughout.

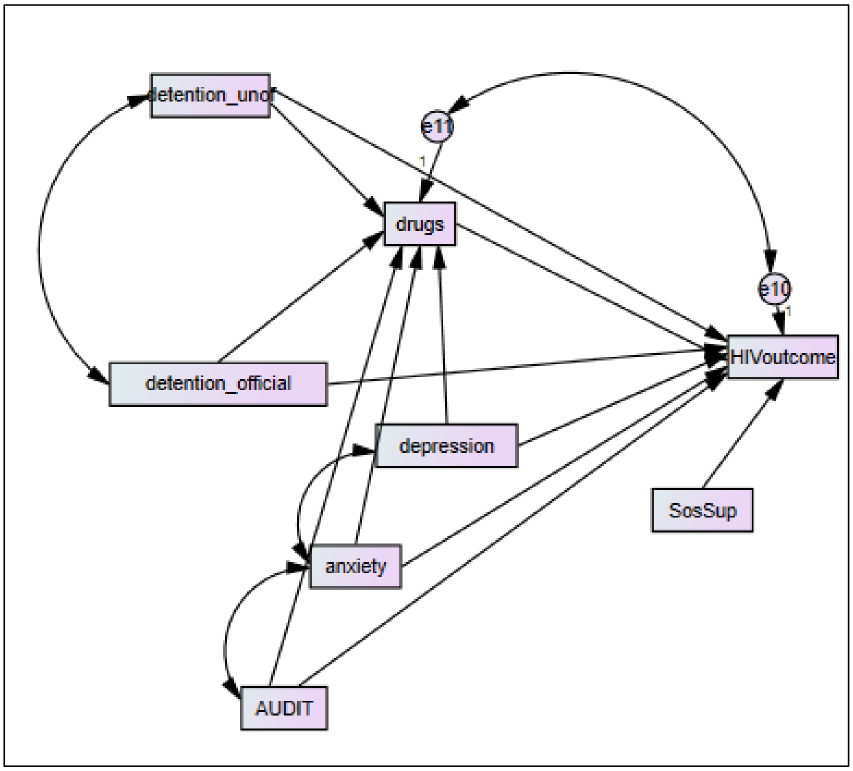

RESULTS

Results are produced by the AMOS text output

*** $p < .05$

Regression Weights:

Predictor	Criterion	B	Beta	S.E.	C.R.	P
Detention unofficial	Drugs	.005	.142	.001	4.221	***
Anxiety	Drugs	.000	.104	.000	2.510	.012
Detention official	Drugs	.002	.052	.001	1.556	.120
Depression	Drugs	.000	.012	.001	.289	.772
AUDIT	Drugs	.001	.160	.000	4.800	***
Social support	Drugs	.001	.027	.002	.824	.410
Drugs	HIV risk	200.967	.553	10.237	19.632	***
Social support	HIV risk	3.413	.195	.479	7.126	***
Detention official	HIV risk	-.931	-.059	.442	-2.106	.035
Detention unofficial	HIV risk	.629	.045	.391	1.609	.108
Depression	HIV risk	-.403	-.091	.151	-2.660	.008
Anxiety	HIV risk	.092	.054	.058	1.587	.113
AUDIT	HIV risk	-.014	-.006	.066	-.212	.832

Covariances and Correlations:

Variables			Estimate	S.E.	C.R.	P	Correlation
Detention unofficial	<-->	Detention official	.328	.059	5.587	***	.194
Anxiety	<-->	AUDIT	-8.873	2.577	-3.442	***	-.095
Anxiety	<-->	Depression	29.503	1.959	15.061	***	.595

Variances:

Variables	Estimate	S.E.	C.R.	P
Detention unofficial	1.930	.093	20.748	***
Social Support	1.212	.058	20.748	***
Anxiety	130.060	6.248	20.815	***
Detention official	1.480	.071	20.748	***
Depression	18.882	.910	20.748	***
AUDIT	67.187	3.238	20.748	***
e11	.003	.000	20.748	***
e10	239.187	11.528	20.748	***

Squared Multiple Correlations (endogenous variables only):

Variables	Estimate
Drugs	.061
HIV risk	.359

Model 2. Base aggregate model (trimmed based on Model 1 results) with covariates for two countries combined with standardized coefficients and measures of fit, and without bootstrap procedure.

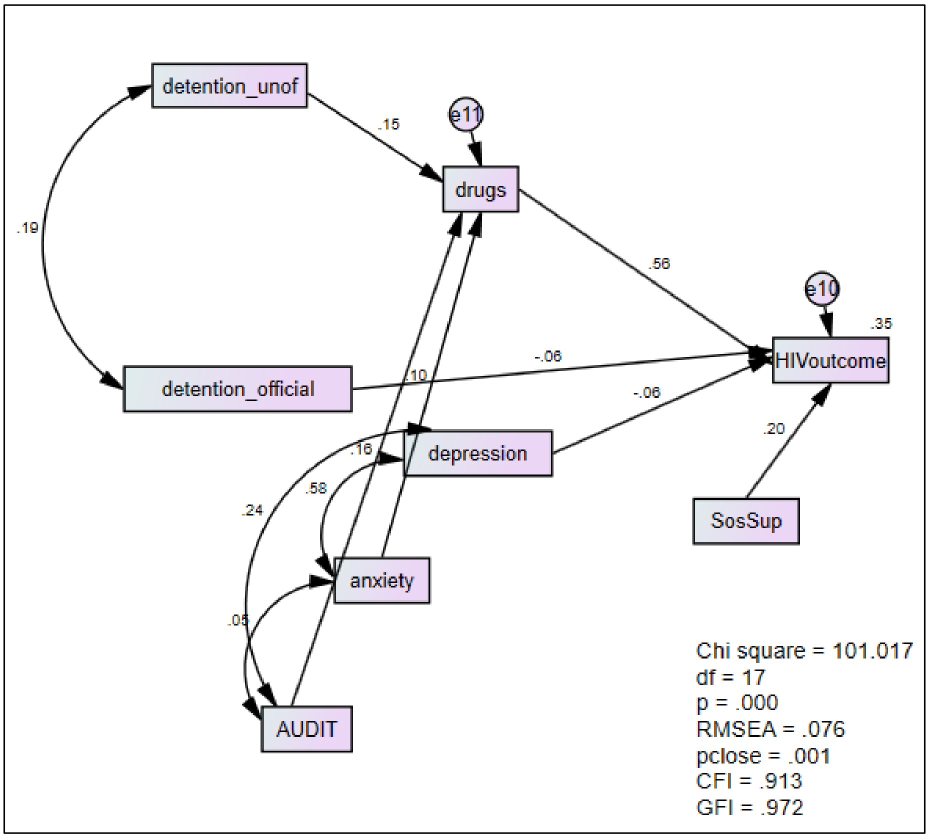

Notes for Model (Default model)

Number of distinct sample moments:	36
Number of distinct parameters to be estimated:	19
Degrees of freedom (36 - 19):	17

Chi-square = 101.017
Degrees of freedom = 17
Probability level = .000

RESULTS

Regression Weights:

Predictor	Criterion	B	Beta	S.E.	C.R.	P
Detention unofficial	Drugs	.006	.152	.001	4.601	***
Anxiety	Drugs	.000	.102	.000	3.090	.002
AUDIT	Drugs	.001	.161	.000	4.882	***
Drugs	HIV risk	204.208	.563	9.980	20.463	***
Social support	HIV risk	3.429	.196	.480	7.142	***
Detention official	HIV risk	-.876	-.055	.435	-2.014	.044
Depression	HIV risk	-.250	-.056	.122	-2.049	.040

Covariances and Correlations:

Variables			Estimate	S.E.	C.R.	P	Correlation
Detention unofficial	<-->	Detention official	.328	.059	5.587	***	.194
Anxiety	<-->	AUDIT	4.225	3.146	1.343	.179	.046
Anxiety	<-->	Depression	28.396	1.927	14.737	***	.581
AUDIT	<-->	Depression	8.383	1.247	6.722	***	.235

Variances:

Variables	Estimate	S.E.	C.R.	P
Detention unofficial	1.930	.093	20.748	***
Social support	1.212	.058	20.748	***
Anxiety	126.601	6.102	20.748	***
AUDIT	67.187	3.238	20.748	***
Detention official	1.480	.071	20.748	***
Depression	18.882	.910	20.748	***
e11	.003	.000	20.748	***
e10	240.542	11.593	20.748	***

Squared Multiple Correlations:

Variables	Estimate
Drugs	.061
HIV risk	.354

Model Fit Summary

Model	GFI	AGFI	CFI	RMSEA	LO 90	HI 90	PCLOSE
Default model	.972	.940	.913	.076	.062	.090	.001
Independence model	.795	.736	.000	.200	.189	.211	.000

Model 3. Base aggregate model (trimmed based on Model 2 results) with covariates for two countries combined, with standardized coefficients, measures of fit, and without bootstrap procedure.

Note: This model is subsequently used for multi-group procedure (Model 4).

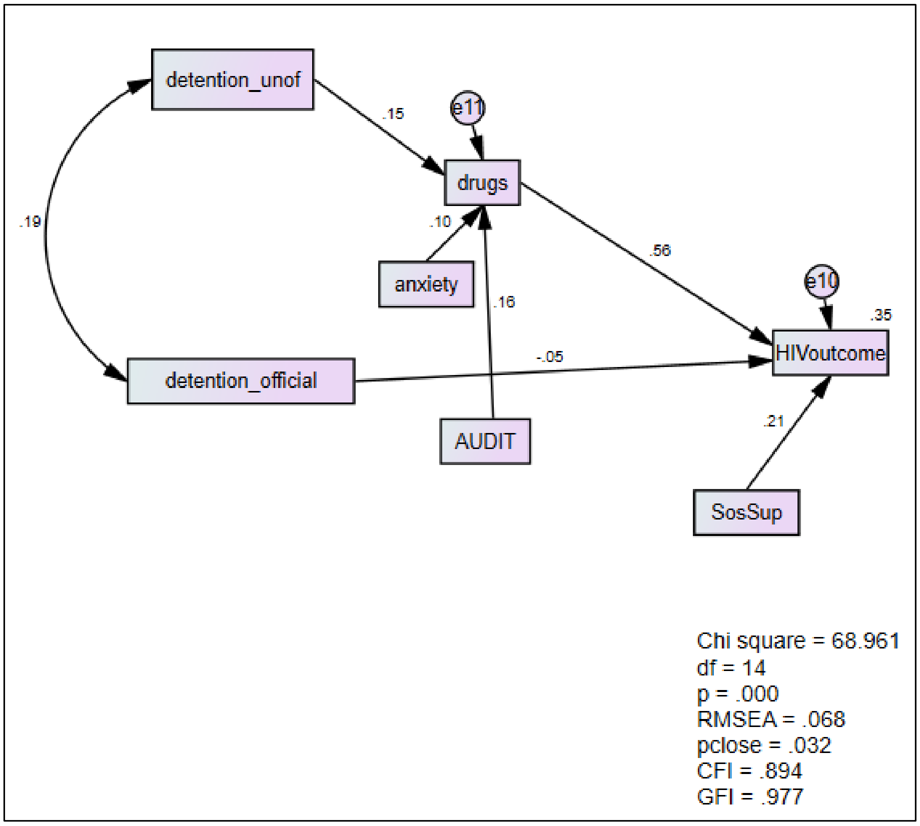

Notes for Model

Number of distinct sample moments:	28
Number of distinct parameters to be estimated:	14
Degrees of freedom (28 - 14):	14

Minimum was achieved
Chi-square = 68.961
Degrees of freedom = 14
Probability level = .000

RESULTS

Regression Weights:

Predictor	Criterion	B	Beta	S.E.	C.R.	P
Detention unofficial	Drugs	.006	.152	.001	4.601	***
Anxiety	Drugs	.000	.102	.000	3.093	.002
AUDIT	Drugs	.001	.161	.000	4.887	***
Drugs	HIV risk	201.713	.556	9.963	20.246	***
Social Support	HIV risk	3.599	.205	.481	7.477	***
Detention official	HIV risk	-.856	-.051	.436	-1.964	.051

Covariances and Correlations:

Variables			Estimate	S.E.	C.R.	P	Correlation
Detention unofficial	<-->	Detention official	.328	.059	5.587	***	.194

Variances:

Variables	Estimate	S.E.	C.R.	P
Detention unofficial	1.930	.093	20.748	***
Social Support	1.212	.058	20.748	***
Anxiety	126.601	6.102	20.748	***
Detention official	1.480	.071	20.748	***
AUDIT	67.187	3.238	20.748	***
e11	.003	.000	20.748	***
e10	241.673	11.648	20.748	***

Squared Multiple Correlations:

Variables	Estimate
Drugs	.060
HIV risk	.352

Model Fit Summary

Model	GFI	AGFI	CFI	RMSEA	LO 90	HI 90	PCLOSE
Default model	.977	.955	.894	.068	.052	.084	.032
Independence model	.858	.810	.000	.170	.157	.182	.000

Model 4 (see the diagram below) is a final Multi-group model with bootstrap and covariates.

Note: Both, Official detention and AUDIT were trimmed from the multi-group indirect effects estimation model because they were no longer significant (once the country was included as a moderator), as shown in the table below for Kyrgyzstan:

Regression Weights: (KYRGYZSTAN)

Predictor	Criterion	B	Beta	S.E.	C.R.	P
Detention unofficial	Drugs	.005	.159	.002	3.101	.002
Anxiety	Drugs	.001	.132	.000	2.576	.010
AUDIT	Drugs	.001	.077	.000	1.502	.133
Drugs	HIV risk	205.05	.651	12.486	16.423	***
Social support	HIV risk	1.766	.078	.901	1.960	.050

Model 4a. Multi-group final model with bootstrap, covariates, and measures of fit for Kyrgyzstan:

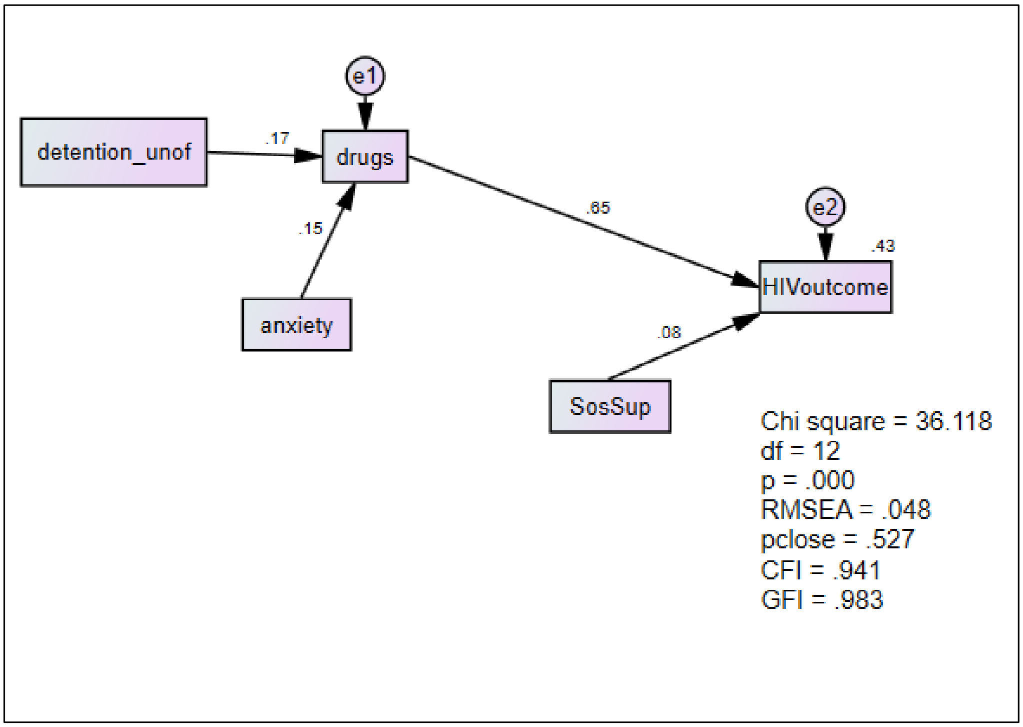

Notes for Model

Number of distinct sample moments:	30
Number of distinct parameters to be estimated:	18
Degrees of freedom (30 - 18):	12

Minimum was achieved
Chi-square = 36.118
Degrees of freedom = 12
Probability level = .000

Model Fit Summary

Model	GFI	AGFI	CFI	RMSEA	LO 90	HI 90	PCLOSE
Default model	.983	.958	.941	.048	.031	.067	.527
Independence model	.850	.774	.000	.154	.142	.167	.000

RESULTS

Regression Weights: (KYRGYZSTAN)

Predictor	Criterion	B	Beta	S.E.	C.R.	P
Detention unofficial	Drugs	.006	.168	.002	3.279	.001
Anxiety	Drugs	.001	.147	.000	2.865	.004
Drugs	HIV risk	205.058	.652	12.442	16.481	***
Social support	HIV risk	1.766	.078	.901	1.960	.050

Variances: (KYRGYZSTAN)

Variables	Estimate	S.E.	C.R.	P
Detention unofficial	4.025	.299	13.475	***
Social support	.915	.068	13.475	***
Anxiety	162.008	12.023	13.475	***
e1	.005	.000	13.475	***
e2	269.855	20.026	13.475	***

Squared Multiple Correlations: (KYRGYZSTAN)

Variables	Estimate
Drugs	.050
HIV risk	.431

Matrices for Total, Direct, and Indirect Effects (KYRGYZSTAN)

Total Effects and Standardized Total Effects (KYRGYZSTAN)

Anxiety		Social support		Detention unofficial		Drugs		
Total	ST	Total	ST	Total	ST	Total	ST	**Criterion**
.001	.147	.000	.000	.006	.168	.000	.000	**Drugs**
.164	.096	1.766	.078	1.188	.109	205.058	.652	**HIV risk**

Direct Effects and Standardized Direct Effects (KYRGYZSTAN)

Anxiety		Social support		Detention unofficial		Drugs		
Total	ST	Total	ST	Total	Total	ST	ST	**Criterion**
.001	.147	.000	.000	.006	.000	.000	.168	**Drugs**
.000	.000	1.766	.078	.000	205.058	.652	.000	**HIV risk**

Indirect Effects and Standardized Indirect Effects (KYRGYZSTAN)

Anxiety		Social support		Detention unofficial		Drugs		
Total	ST	Total	ST	Total	ST	Total	ST	**Criterion**
.000	.000	.000	.000	.000	.000	.000	.000	**Drugs**
.164	.096	.000	.000	1.188	.109	.000	.000	**HIV risk**

Model 4b. Multi-group final model with bootstrap, covariates, standardized coefficients, and measures of fit for Azerbaijan

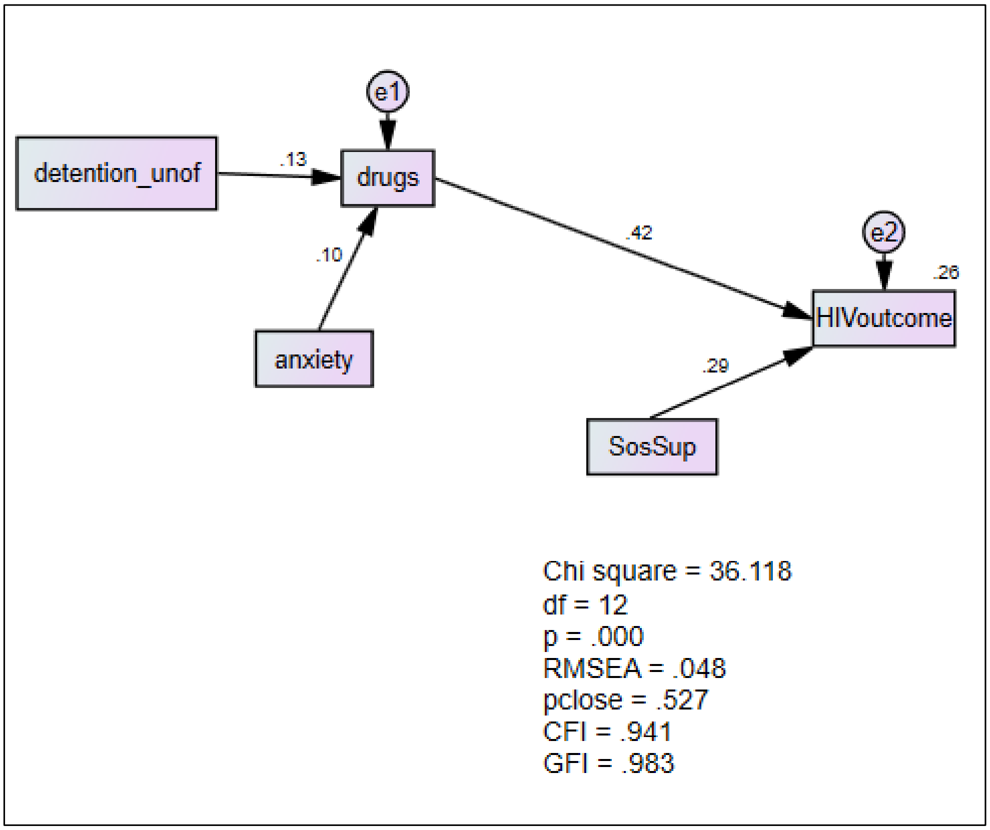

RESULTS

Regression Weights: (AZERBAIJAN)

Predictor	Criterion	B	Beta	S.E.	C.R.	P
Detention unofficial	Drugs	.011	.131	.004	2.952	.003
Anxiety	Drugs	.000	.105	.000	2.369	.018
Drugs	HIV risk	192.51	.417	17.828	10.799	***
Social support	HIV risk	4.279	.292	.566	7.561	***

Variances: (AZERBAIJAN)

Variables	Estimate	S.E.	C.R.	P
Detention unofficial	.212	.013	15.761	***
Social support	1.382	.088	15.761	***
Anxiety	71.583	4.542	15.761	***
e1	.001	.000	15.761	***
e2	219.900	13.952	15.761	***

Squared Multiple Correlations: (AZERBAIJAN)

Variables	Estimate
Drugs	.028
HIV risk	.259

Matrices for Total, Direct, and Indirect Effects (AZERBAIJAN)

Total Effects and Standardized Total Effects (AZERBAIJAN)

Anxiety		Social support		Detention unofficial		Drugs		
Total	ST	Total	ST	Total	ST	Total	ST	**Criterion**
.000	.105	.000	.000	.011	.131	.000	.000	**Drugs**
.089	.044	4.279	.292	2.035	.054	192.518	.417	**HIV risk**

Direct Effects Standardized Direct Effects (AZERBAIJAN)

Anxiety		Social support		Detention unofficial		Drugs		
Total	ST	Total	ST	Total	ST	Total	ST	**Criterion**
.000	.105	.000	.000	.011	.131	.000	.000	**Drugs**
.000	.000	4.279	.292	.000	.000	192.518	.417	**HIV risk**

Indirect Effects and Standardized Indirect Effects (AZERBAIJAN)

Anxiety		Social support		Detention unofficial		Drugs		
Total	ST	Total	ST	Total	ST	Total	ST	**Criterion**
.000	.000	.000	.000	.000	.000	.000	.000	**Drugs**
.089	.044	.000	.000	2.035	.054	.000	.000	**HIV risk**

Note: This Final model above is reported in Figures 1 (aggregate with no covariates) and table 2 (multi-group with indirect, direct, and total effects) of the originally submitted manuscript. Significant covariates from the results presented above are reported in table 6 of the manuscript.

Model 5. KYRGYZSTAN WPDI with two detention variables. The identical path diagram is also provided in the originally submitted appendixmanuscript (Model Figure 2), and the results are summarized in Table 7.

Note: WPDI model presented in the manuscript (Figure 2) combines Unofficial and Official detention.

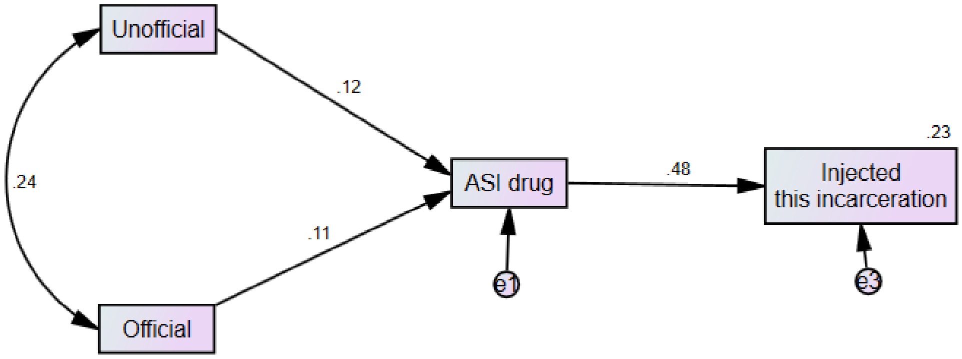

Notes for Model

Number of distinct sample moments:	10
Number of distinct parameters to be estimated:	8
Degrees of freedom (10 - 8):	2

Minimum was achieved
Chi-square = .435
Degrees of freedom = 2
Probability level = .805

Model Fit Summary

RMR, GFI

Model	GFI	AGFI	CFI	RMSEA	LO 90	HI 90	PCLOSE
Default model	.999	.997	1.000	.000	.000	.066	.911
Independence model	.852	.753	.000	.239	.204	.276	.000

RESULTS

Regression Weights:

Predictor	Criterion	B	Beta	S.E.	C.R.	P
Detention unofficial	Addiction severity	.003	.120	.001	2.219	.027
Detention official	Addiction severity	.008	.113	.004	2.103	.036
Addiction severity	WPDI	2.228	.481	.216	10.302	***

Covariances and Correlations:

Variables			Estimate	S.E.	C.R.	P	Correlation
Detention unofficial	<-->	Detention official	.944	.213	4.434	***	.243

Variances:

Variables	Estimate	S.E.	C.R.	P
Detention unofficial	11.578	.871	13.285	***
Detention official	1.306	.098	13.285	***
e1	.007	.001	13.285	***
e3	.119	.009	13.285	***

Squared Multiple Correlations:

Variables	Estimate
Addiction severity	.034
WPDI	.231

Matrices for Total, Direct, and Indirect Effects

Total Effects and Standardized Total Effects

Detention official		Detention unofficial		Addiction severity		
Total	ST	Total	ST	Total	ST	**Criterion**
.008	.113	.003	.120	.000	.000	**Addiction severity**
.019	.055	.007	.058	2.228	.481	**WPDI**

Direct Effects and Standardized Direct Effects

Detention official		Detention unofficial		Addiction severity		
Total	ST	Total	ST	Total	ST	**Criterion**
.008	.113	.003	.120	.000	.000	**Addiction severity**
.000	.000	.000	.000	2.228	.481	**WPDI**

Indirect Effects and Standardized Indirect Effects

Detention official		Detention unofficial		Addiction severity		
Total	ST	Total	ST	Total	ST	**Criterion**
.000	.000	.000	.000	.000	.000	**Addiction severity**
.019	.055	.007	.058	.000	.000	**WPDI**

References

1. Preacher KJ, Rucker DD, Hayes AF. Addressing Moderated Mediation Hypotheses: Theory, Methods, and Prescriptions. *Multivariate Behavioral Research.* 2007;42(1):185-227.
2. Bollen KA, Stine RA. Bootstrapping goodness-of-fit measures in structural equation models. *Sociological Methods & Research.* 1992;21(2):205-229.
3. Hu L-t, Bentler PM. Fit indices in covariance structure modeling: Sensitivity to underparameterized model misspecification. *Psychological methods.* 1998;3(4):424.
4. Bentler PM. Comparative fit indexes in structural models. *Psychological bulletin.* 1990;107(2):238.
5. Kline RB. *Principles and practice of structural equation modeling.* Guilford publications, New York, NY,; 2015.
6. Hoyle RH. Path analysis and structural equation modeling with latent variables. In: Cooper H., ed. *APA handbook of research methods in psychology: Vol. 2. Quantitative, qualitative, neuropsychological, and biological*. American Psychological Association, Washington, DC; 2012:333-367.

Formaterat: Teckensnitt:Kursiv
